# Supplementary material for: Phenotype selection due to mutational robustness
Source: PLoS One. 2024 Nov 18;19(11):e0311058. doi: 10.1371/journal.pone.0311058 (PMC11573163; doi:10.1371/journal.pone.0311058)
Supplement: S1 Fig — The red lines with arrowhead indicate activation and the blue lines with barhead indicate repression. (PDF) [file pone.0311058.s001.pdf]

S1 Fig

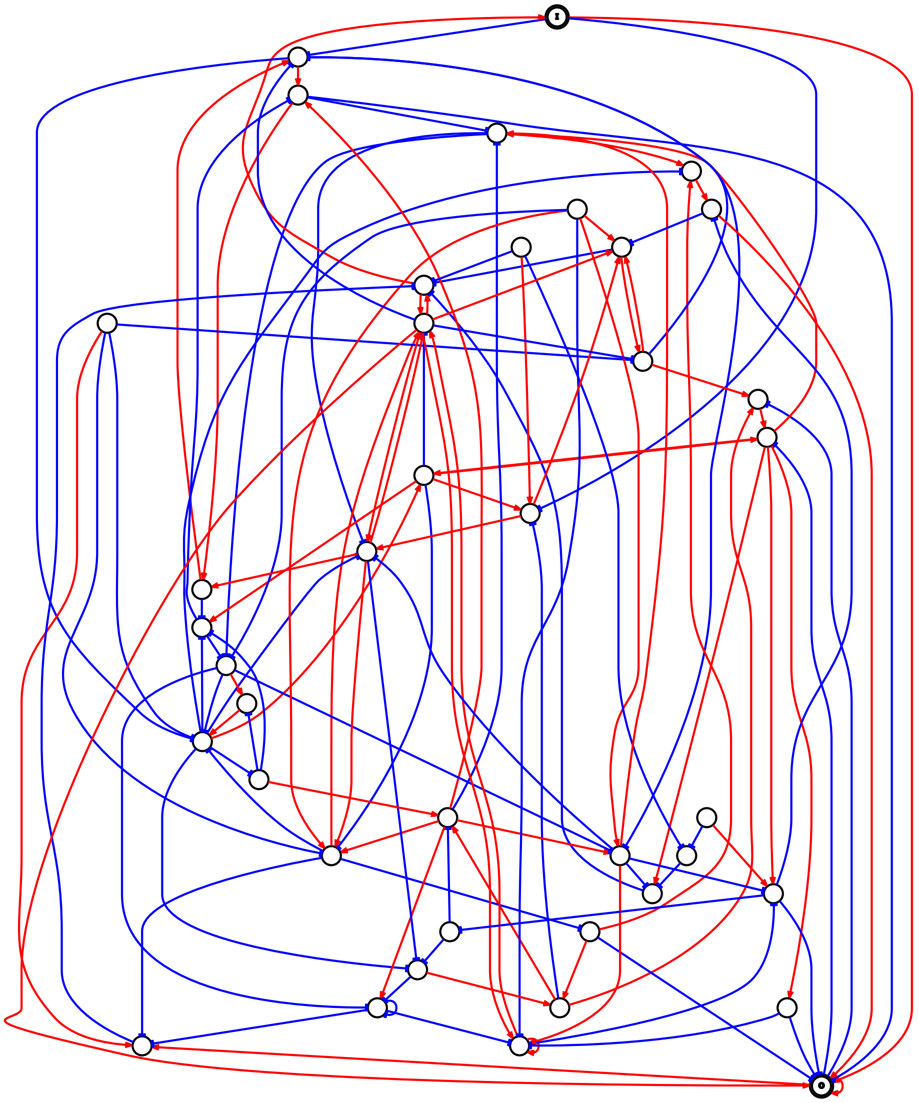

One-way switch GRN with the maximum number of essential edges, $N_{ee} = 109$ obtained by McMC. The red lines with arrowhead indicate activation and the blue lines with barhead indicate repression.
