# Supplementary material for: Phenotype selection due to mutational robustness
Source: PLoS One. 2024 Nov 18;19(11):e0311058. doi: 10.1371/journal.pone.0311058 (PMC11573163; doi:10.1371/journal.pone.0311058)
Supplement: S2 Fig — The red lines with arrowhead indicate activation and the blue lines with barhead indicate repression. (PDF) [file pone.0311058.s002.pdf]

S2 Fig

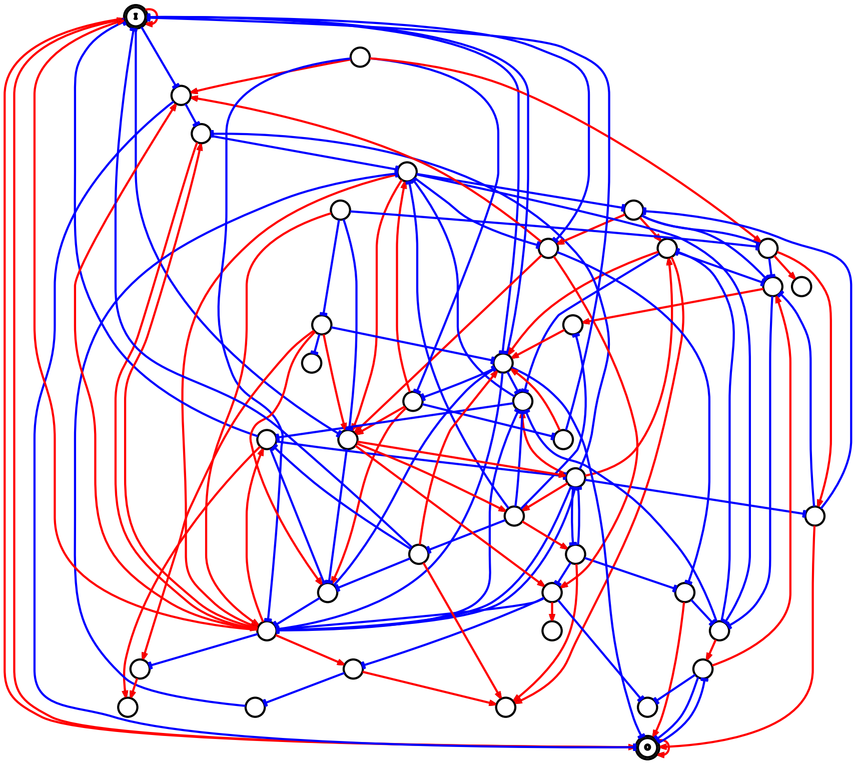

An example from 42 one-way switch GRNs having no essential edge obtained by ESr. The red lines with arrowhead indicate activation and the blue lines with barhead indicate repression.
