# Supplementary material for: Phenotype selection due to mutational robustness
Source: PLoS One. 2024 Nov 18;19(11):e0311058. doi: 10.1371/journal.pone.0311058 (PMC11573163; doi:10.1371/journal.pone.0311058)
Supplement: Attachment — Submitted filename: Reply_to_reviewers.pdf [file pone.0311058.s003.pdf]

Reply to reviewer #1

The author presents a study of gene regulatory networks (GRNs), where the presence of three kinds of dynamical systems behavior are compared between an unbiased sampling of the genotype space (using a Monte Carlo approach) and two kinds of evolutionary simulations. The main finding is that evolution strongly selects against one-way behavior, whilst mono and toggle-switch behavior appear to be favored. The author argues that one-way behavior is less robust than the other two behaviors, in the sense that one-way tends to require more essential edges.
I applaud the massive amount of simulations done to establish a set of 50k lineages to analyze, and the innovative application of McMC sampling to generate unbiased probability distributions of the three kinds of dynamical systems behavior. The results are not unanticipated, as it is known that (long-term) evolution in a constant environment favors the evolution of robustness.
Overall, the study has been well done and is a nice contribution to the (in silico) evolutionary biology community. In my opinion, it is worthy of publication in PLoS ONE after revisions. Please find below my comments.

Thank you very much for your effort in carefully reading the manuscript. Your advice was constructive to improve the manuscript. I considered your comments fully and revised the manuscript accordingly, as described below.

citations are put after the sentence; they should be within, i.e. before the end-of-sentence mark.

I changed the position of citations.

please consider having the manuscript checked by a native English person. Especially the use of articles (the) is problematic (too abundant) and hinders a smooth reading experience.

I consulted an English editing service and rewrote the manuscript accordingly. I put the certificate at the end of this letter.

in the Introduction, it is argued that selection for robustness is almost independent of fitness (line 17). What does the author mean? That there is a trade-off between being fitter and being more robust? I do not remember the precise works that study this relationship, but surely there is such a trade-off and I would expect it to depend on selection pressure.

My former argument was too strong. So, I changed the corresponding sentence as follows:

> To emphasize the fact that this selection bias is different from one determined directly by fitness, this bias is called “second-order”

> in the Introduction, the author states that second order selection enhances evolvability (line 18-19). Since evolvability is such a loosely used term, it would be good if the author defines what exactly they mean in this context, e.g. by choosing one of the three definitions from Pigliucci. It seems, the first definition, evolvability as heritability is the one.

I agree that evolvability has been used in many different ways. Referring to the review by Pigliucci (Nat Rev Gen), I think the second concept in the list by Wagner and Altenberg is appropriate in the present context. I added the following sentence at the end of the paragraph:

> The word “evolvability” has been used in many ways; here we used it in the manner of Wagner and Altenberg, namely, the ability to make emergence of new traits or new phenotypes.

I added two papers, Pegliucci (Nat Rev Gen) and Wagner and Altenberg (Evolution), to the citation.

> in the Introduction, the author argues that fitness traditionally is expressed as the number of offspring. I agree, this is surely the case in population genetics. In many simulation studies, however, fitness is a value computed as the author does. So, it is not that special and I think it would be good if the text reflected this more clearly.

I change the paragraphs as follows:

> We note here the usage of the word ”fitness”. In population genetice, fitness is defined based on the number of offspring, and the effects of all the selection biases are taken into account. In this paper, however, we define fitness by a value calculated from a specific fitness function and consider selection biases separately. This definition is widely used in evolutionary simulations.

> line 106 could be put in Eq. 3 for completeness: $f = max(x_out(1) - x_out(0), 0)$

I changed the definition of fitness accordingly.

> in the Methods, lines 107-119 and lines 120-127 read more like results than Methods. The author should consider moving the text.

I moved a part of the paragraphs to the ”Classification of Phenotypes” subsection, a part to the ”Results” section, and the rest to the ”Discussion” sec-

tion. The figures were moved to the "Results" section. Please see the marked manuscript.

> in line 136 it is not clear what happens to the sign of an edge (activation, inhibition) as it is rewired.

I added the following sentence:

> The sign of the new edge was selected randomly.

> in the Methods, the explanations of ES0 and EST are confusing. The zero in ES0 has nothing to do with the temperature beta of EST, whilst I expected these two to be related. And naming EST explicitly as "stochastic" (line 146) made me think the other one is not... which is not the case, of course, since mutations always introduce some stochasticity. Alternatively, one may argue that "sure mutations are stochastic", but the network is computed in a deterministic manner, so fitness is deterministic and taking the top half of the population is not stochastic either. That is fine with me, but it would be good to make that more explicit, f.i. by stating that here we talk about the selection step only. Some rephrasing is necessary here. Also, at some point I thought EST was a continuation of ES0, but that appears not to be the case either (at least not in operational terms, conceptually yes?). So, do I understand it correctly that ES0 has a classical selection scheme of replacing the bottom-half of the population? And that EST is more like a noisy propertional-fitness selection scheme? I think it would be better either to explain how the two selection schemes are unified (since right now their naming suggests there is a single framework behind) or to choose two really different names, like ES_half and ES_noise, to acknowledge the independent approaches.

The mutation is random in both methods. The differences between the two methods are only in the selection method that evolution temperature was introduced in EST, and the network is selected randomly with bias by fitness. So, as you pointed out, both methods contain stochastic parts. I changed the abbreviations from ES0 to ESh (h stands for high) and from EST to ESr (r stands for random) and changed the beginning of the method description as follows:

> We conducted two types of ES: ESh (where h represents "high fitness") and ESr (where r represents "randomness in selection"). These two methods differ in the selection of genotypes, whereas the generation of new genotypes is the same.

in lines 188 and on, essential edges are defined in a way that evolution never experiences, which may impact the results reported. I mean: removing an edge is normally accompanied by the addition of a new one somewhere else in the network (the definition of a mutation). So to measure mutational robustness by subjecting networks to modifications that are not mutations and that the GRNs never evolved with is inconsistent. It is likely to be rather harmless, but I think this needs to be checked or at least reported as a caveat.

I agree that the mutation procedures are different between simulations and counting the essential edge, and it may cause some discrepancies. To confirm the appropriateness of counting essential edges to evaluate mutational robustness, I conducted random rewiring experiments of an edge (the same mutation method as simulations) 1000 times for 10000 GRNs at $f \in [0.9, 0.91]$. Please see the new Fig 2 for the results. The ratio of lethal mutations and the number of essential edges showed a good positive correlation. Thus, the number of essential edges is a good indicator of mutational robustness.

I added the following sentences to explain this:

> The mutation procedure conducted in evolutionary simulations is not a single-edge cut but a single-edge rewiring. Then, we took 10000 GRNs with fitness $f \in [0.9, 0.91]$ obtained by McMC, and attempted random single-edge rewiring 1000 times for each GRN to investigate the ratio of lethal mutations, that is, the ratio of mutations that reduced f to below 0.5. The results are shown in Fig 2. The number of essential edges N_{ee} and the ratio of lethal mutations r_l exhibit a strong positive correlation, although it is slightly upward convex. Therefore, N_{ee} is a good indicator of mutational robustness.

in the Results, the author does not elaborate on any patterns of network structure that evolution may have come up with. How many edges are activating / inhibiting in evolved and sampled networks? Is the node in- and outdegree of evolved networks very different from the sampled ones? Are there many forcing structures (similar to the AND and OR functions in Boolean networks)? Understanding such structural patterns may give deeper insight into what evolution prefers as a solution to the task of reacting to an input signal.

I analyzed network structures and their dynamical properties for mutationally robust GRNs obtained by ESr and highly non-robust GRNs obtained by McMC. I found that these two sets of GRNs bear quite different properties. While the robust GRNs contain a simple motif, non-robust GRNs do not. I conducted a detailed analysis of the network properties of these two sets. The results are described in a new "Network properties" subsection. As this subsection is long and accompanied by many figures, please read the manuscript. I hope these

new analyses satisfy your requirements.

> Discussion: this study focuses on the "classic study case" of evolution in a constant environment, where a single task needs to be done. There are many other settings that have been left unexplored (e.g. changing environments, multiple tasks, co-evolution, allowing for changes in nr of genes & nr of edges), which makes me rather hesitant at the claim of universality in the Discussion. I know "universality" has a specific meaning in physics, yet I fear a biology or comp sci colleague could easily misunderstand it. I would suggest the author to either not claim it, or better contextualize the claim. Perhaps there are specific experimental results that could be mentioned (E. coli, yeast)? Note that I do not suggest to do more simulations under different kinds of evolutionary scenarios!

I agree that universality is a too strong word. I changed the sentences as follows:

> This phenotype selection can occur whenever more than one phenotype exists for the same fitness value; thus, we expect this phenomenon to occur in broad situations in real living systems Therefore, mutational robustness may play an essential role in the evolution of certain phenotypes

I also looked for related experimental papers, and found that Maeda et al. (Plos Biol) reported multiple peaks in the fitness landscape of E.Coli under existence of some antibiotics. It means the phenotype selection we proposed may occur in this system, although experimental confirmation will be difficult. I added the following sentences:

> Multiple peaks in the fitness landscape of *Escherichia coli* have been experimentally observed in the presence of antibiotics [37]. This implies that more than one phenotype has similar fitness. Under such circumstances, phenotype selection due to the present scenario may occur, although this may be difficult to confirm.

> I don't understand why the reported results are third-order. I thought of them as the mechanism (or implementation) that evolution selected. Mutational robustness must manifest in some way. Iow, for me they are second-order.

I deleted the mention on third-order.

> it would be good to discuss limitations of the study: besides the above mentioned constant environment, a fixed nr of genes and a fixed total nr of edges is bound to have had an impact on the results presented here.

I added the following paragraph:

> The present study focused on limited situations, such as a constant environment, a fixed number of genes and edges, and a fixed number of populations in evolutionary simulations. Extending this study to other situations is left for future work.

the final statement that GAs explore in a biased way the optimization landscape is common knowledge. I would encourage the author to come up with a stronger concluding sentence at the end of their manuscript.

I modified the Discussion section, and deleted the mention on GA.

line 2: ”developed” is not the term to use, since in biology development is rather different from evolution.

I changed the first sentence as follows:

> Living systems in their present form have been created through Darwinian evolution, a process in which a genotype changes so that the phenotype fits the environmental conditions.

in figure 1 colors are not explained, especially those of the edges.

I added explanation for colors of edges in the caption.

it would be helpful to provide titles in the panels in figures 4 and 6 with ”mono”, ”toggle” and ”one-way” and similarly, in figure 5 with ”McMC” and ”EST”. Otherwise, one keeps going back and forth between caption and figure.

I added titles to many figures.

in many panels, errorbars are difficult to see because they overlap with the symbol. Also, too many symbols together make for very fat lines, could the author space them better? Smaller symbols? Less symbols?

I redrawn most of the figures using thinner lines and smaller symbols. For visual clarity, I changed square symbols to triangular symbols.

N_e is a famous technical term/symbol in evolutionary biology meaning ”effective population size”. I advice the author to choose a different one for ”nr of essential edges” to avoid confusion. Already $N_e e$ could work, not?

I changed N_e to $N_e e$.

editage

Editing Certificate

This document certifies that the paper listed below has been edited to ensure that the language is clear and free of errors. The logical presentation of ideas and the structure of the paper were also checked during the editing process. The edit was performed by professional editors at Editage, a division of Cactus Communications. The intent of the author's message was not altered in any way during the editing process. The quality of the edit has been guaranteed, with the assumption that our suggested changes have been accepted and have not been further altered without the knowledge of our editors.

MANUSCRIPT TITLE

Phenotype selection due to mutational robustness

AUTHORS

Macoto Kikuchi

ISSUED ON

August 23, 2024

JOB CODE

QOYVE_1

THE EDITAGE Promise QUALITY GUARANTEE

Prabh Grewal
Senior Vice President - Editage

editage helping you get published

Since 2002, Editage has helped over 430,000 authors publish around 1.2 million research papers in scholarly journals across over 1000 disciplines through editorial, translation, transcription, and publication support services. Editage is a brand of Cactus Communications (cactusglobal.com), a science communication and technology company.

CACTUS.

GLOBAL :
+1(833) 979-0061 | request@editage.com

JAPAN :
0120-50-2987 | submissions@editage.com
